# An Uncommon Osseous Frontal Sinus Tumor: Monostotic Paget's Disease

**DOI:** 10.1155/2013/650428

**Published:** 2013-12-07

**Authors:** Varant Labajian, Evie Landry, Fahad Alfawwaz, Chi Lai, Shaun J. Kilty

**Affiliations:** ^1^Department of Otolaryngology-Head and Neck Surgery, The Ottawa Hospital, Civic Campus, Parkdale Clinic, Room 459, 737 Parkdale Avenue, Ottawa, ON, Canada K1Y 1J8; ^2^Department of Pathology and Laboratory Medicine, The University of Ottawa, Ottawa, ON, Canada; ^3^The Ottawa Hospital Research Institute, Ottawa, ON, Canada

## Abstract

Paget's disease of the bone is a disorder characterized by abnormal breakdown and formation of bone tissue. The cause is believed to be either viral or genetic in origin. Most of the time, patients are asymptomatic. Diagnosis is supported by findings from multiple modalities including serum markers, imaging, bone scans, and histology. We present a rare case of Paget's disease of the bone involving the frontal sinus. We review the relevant clinical, diagnostic, and histological findings. We also suggest indications for the management of monostotic Paget's disease of the frontal sinus.

## 1. Introduction

Paget's disease of the bone (PDB), first described in 1877 by the English surgeon Sir James Paget, has been defined as a disorder of bone remodeling. Characteristically, PDB begins with accelerated bone resorption followed by compensatory bone formation leaving behind a structurally disorganized bone matrix. The disease is most prevalent in western Europe, where it affects more men than women (1.8 : 1) and it is most commonly diagnosed after the fifth decade of life [1].

The diagnosis of PDB can be made based on multiple modalities including elevated serum alkaline phosphatase, urinary markers, radiologic findings, radionuclide bone scans, and pathology. PDB involves a single bone (monostotic) in 10–35% of cases, but a multifocal (polyostotic) presentation affecting 2 or more bones is more common. Although the disease can affect any bone in the body, PDB has a predilection for the axial skeleton with skull involvement being the fifth in order of frequency [1]. We report an extremely rare case of PDB of the frontal sinus.

## 2. Case Report

**Figure 1:**
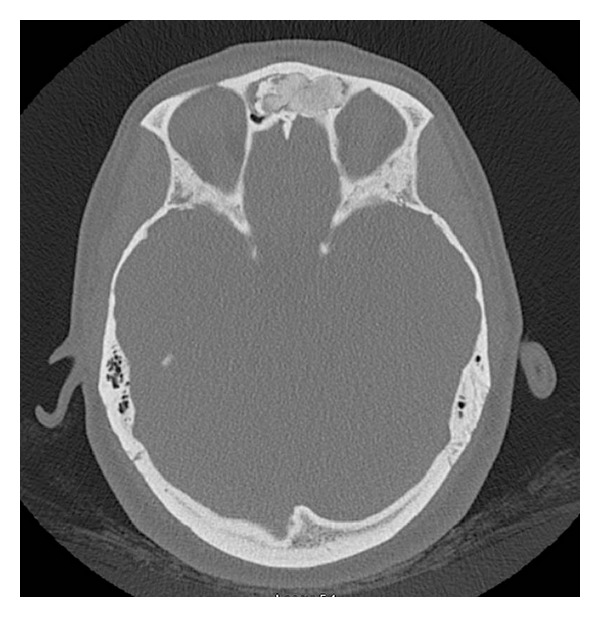
Axial CT scan showing the osseous tumor filling the frontal recess bilaterally.

**Figure 2:**
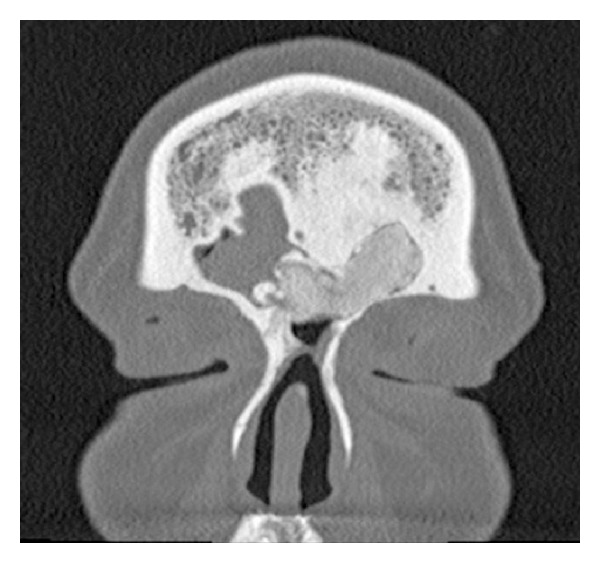
Coronal CT scan showing the osseous mass filling the left frontal sinus and extending into the right.

**Figure 3:**
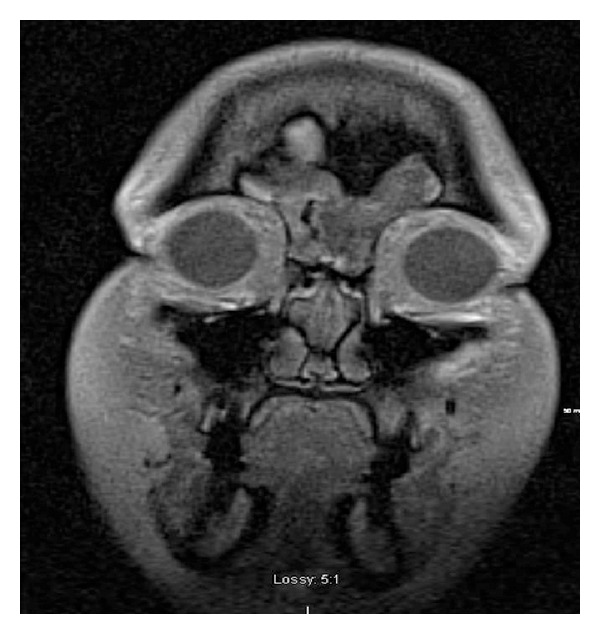
Coronal T1 weighted MRI showing the osseous mass filling the left frontal sinus and extending into the right.

A 39-year-old female was seen in consultation after having presented to the emergency department with complaints of left-sided headache and left facial paresthesias. A head computed tomography (CT) scan revealed an osseous mass in the left frontal and anterior ethmoid sinus extending into the right frontal sinus (Figures 1 and 2). An MRI of the brain identified a left-sided frontal sinus tumor that was benign in appearance and of bone origin (Figure 3). Radiologic impression was a fibro-osseous lesion either representing a fibrous dysplasia or an atypical osteoma. On examination, the patient had no facial asymmetry with no facial nerve dysfunction and she reported some paresthesias on the left side on sensory exam in the V1 distribution. Flexible nasal endoscopy was normal on the right and showed a slightly irregular edematous mucosa in the left superoanterior surface of the bulla ethmoidalis. A specific mass was not identified.

The patient had undergone an incomplete endoscopic resection of the osseous lesion one year previously due to an inability to address the posterior attachment of the lesion to the skull base. Furthermore, the specimen from that procedure did not provide a pathologic diagnosis. Nonetheless, the patient's headache did improve for several months following that procedure. However, by the time of presentation now, she had six months of progressively worsening left-sided headache despite both topical and systemic treatments for sinus disease and medical management for her headaches.

**Figure 4:**
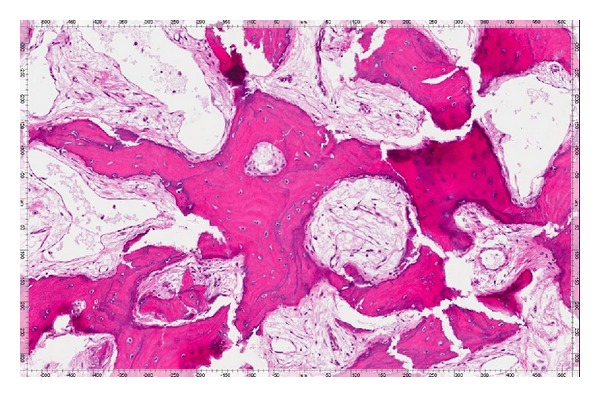
Medium power (100X, H&E stain) photomicrograph demonstrates thickened bone trabeculae exhibiting prominent reversal lines and increased vascularity within the intertrabecular spaces.

**Figure 5:**
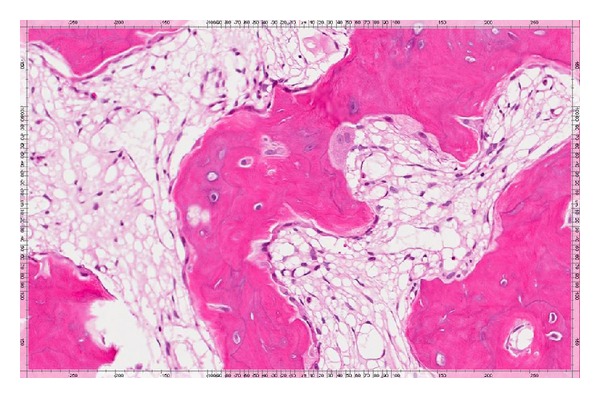
At higher magnification (200X, H&E stain), osteoblastic rimming of bone trabeculae is notably present with focal osteoclastic activity. There are also several resorptive surfaces in addition to reversal lines.

The patient was ordered a CT scan of the paranasal sinuses that showed significant interval growth of the osseous mass since the scan prior to her endoscopic procedure. The mass now crossed the midline into the right frontoethmoidal recess. Given the inability to attain symptom control with medical treatment for this patient, surgical options were considered. After discussing the surgical options, the patient consented to proceed with an open bicoronal osteoplastic flap approach to the frontal sinus in order to access the full extent of the lesion bilaterally and remove it from its extensive attachment to the anterior skull base. This was completed without CSF leak or other complications. The patient was hospitalized for one night and was discharged the following day. She had immediate relief from her longstanding left frontal headache. Pathologic analysis of the specimen was completed and it revealed a bone tumor with a histological appearance consistent with PDB. Both burnt-out and focally active forms of PDB were identified (Figures 4 and 5).

**Figure 6:**
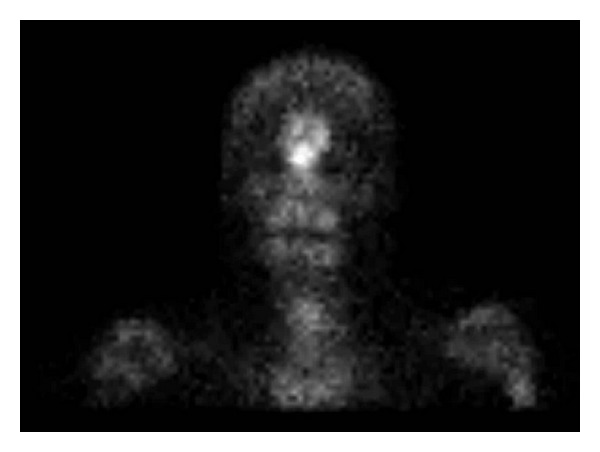
Bone scan showing increased uptake in the frontal sinus.

Postoperatively, the patient did extremely well with complete resolution of her symptoms. Given the pathologic diagnosis, she was referred to a rheumatologist for further evaluation for PDB. A whole-body bone scan with (single-photon emission computed tomography) SPECT demonstrated abnormal bony uptake in the left frontal bone in keeping with postsurgical changes (Figure 6). An alkaline phosphatase level was drawn and it was within normal limits. The staff rheumatologist's impression was monostotic PDB of the skull inactive and no additional therapy was indicated. The patient is now 8 months postoperatively and symptom free. There were no long-term sequelae from the bicoronal approach and osteoplastic flap.

## 3. Discussion

Paget's disease of the bone involving the paranasal sinuses is extremely rare. There are only a few case reports in the literature. Mckee et al. reported a case of simultaneous mucoepidermoid carcinoma and Paget's disease involving the maxillary sinus [2]. Woo and Schwartz described a case of polyostotic disease where there was involvement within the maxilla [3]. Lee et al. described Paget's disease in the sphenoid sinus with simultaneous mucocele [4]. Lastly, Goto et al. wrote the only publication that has described PDB involving the frontal sinus and in their case there was resultant mucocele formation [5]. In summary, this disease uncommonly presents as isolated frontal sinus disease.

In fact, PDB is usually an asymptomatic process that is discovered incidentally on a radiographic study. However, less commonly patients may present with symptoms, as did this patient, with pain being the most common presenting complaint. Other symptoms are related to the growth of Pagetic bone causing fractures, sinusitis, and intracranial extension, among others. Malignant neoplasm is rarely observed; however, there are reported incidences of osteosarcoma transformation in roughly 1% of cases of PDB. Potential complications of Paget's disease include cardiovascular compromise secondary to high output cardiac states caused by arteriovenous shunting from the increased vascularity seen in Pagetic bone. However, heart failure is not commonly seen unless an underlying cardiac pathology already exists. Other cardiac compromises associated with PDB include vascular calcification and calcified aortic stenosis [6].

Diagnosis of Paget's disease is typically made by radiological modalities, biochemical markers, and pathology. Radiological findings of CT or plain films might show a lesion with cortical thickening, coarse trabecular markings, and sclerotic changes [6]. Radionuclide bone scans are typically performed to rule out active disease elsewhere in the body. Biochemical markers that are routinely tested include ALP and serum or urine telopeptide. The degree of ALP rise usually parallels the degree of active bone resorption and reformation. Thus, a normal ALP value does not necessarily mean that the osseous lesion may not represent Paget's disease, rather it reflects that the disease is in an inactive phase.

There are 3 pathologic phases of the disease. The first phase includes an increased osteolytic phase. The second phase includes active bone formation where lamellar bone is replaced with disorganized woven bone. Finally, the third phase is a sclerotic phase where there is a decrease in the resorption and reformation of bone which leads to a hard, less vascular Pagetic bone also called “burnt-out” phase of PDB which was clearly observed in this patient's pathology (Figure 5) [6].

Treatment of PDB falls in 2 general categories, medical and surgical. First-line medical treatment approved for Paget's disease includes oral bisphosphonates. They suppress bone resorption by secondarily reducing bone formation. Second-line medical treatment includes calcitonin injections which have been shown to reduce the levels of ALP up to 50 percent [6].

Surgical treatment is also considered in all patients with PDB. Since there is a paucity of data for the management of monostotic Paget's disease of the frontal sinus, we believe that indications for surgical management need to be identified. If asymptomatic, nonobstructive to the frontal sinus and without serologic abnormality, this should be closely followed for interval growth and disease progression. Suggested indications for surgical removal include the following: (1) evidence of frontal sinus obstruction leading to symptoms of sinusitis or the development of a mucocele, pneumatocele, or abscess and local compression causing pain; (2) more than 50 percent of the frontal sinus is occupied by the lesion; (3) a lesion in the frontal sinus with extension that is intracranial, intraorbital, or causing significant cosmetic deformity of the anterior wall of the frontal sinus. This patient's CT scan clearly shows involvement of the frontoethmoidal recess and the lesion was posteriorly based whilst the patient was symptomatic of disease.

## 4. Conclusion

Monostotic Paget's disease of the frontal sinus is rare. However, it should be considered as part of the differential diagnosis of an isolated osseous lesion in the frontal sinus. The presented case illustrates the important clinical, radiologic, and pathologic findings of monostotic Paget's disease and suggests indications for its surgical management.
